# The relationship between psychopathy and autism: a systematic review and narrative synthesis

**DOI:** 10.3389/fpsyt.2024.1375170

**Published:** 2024-03-27

**Authors:** Kate Maguire, Hayley Warman, Frances Blumenfeld, Peter E. Langdon

**Affiliations:** ^1^ School of Health and Social Care, University of Essex, Colchester, United Kingdom; ^2^ Centre for Research in Intellectual and Developmental Disabilities (CIDD), University of Warwick, Coventry, United Kingdom; ^3^ Coventry and Warwickshire Partnership National Health Service (NHS) Trust, Coventry, United Kingdom; ^4^ Worcestershire Health and Care National Health Service (NHS) Trust, Worcester, United Kingdom

**Keywords:** autism, psychopathy, callous unemotional traits, review - systematic, narrative synthesis

## Abstract

**Background and methods:**

The aim of this systematic review was to synthesise research examining the relationship between autism and psychopathy to: (a) better understand the relationship between these two constructs, and (b) describe the clinical manifestation of the two when they co-occur. A systematic search of the literature returned 36 studies.

**Results:**

Across all ages, autistic individuals and those with elevated autistic traits but no autistic diagnoses appeared to have increased callous and unemotional traits or psychopathy relative to the general population. Several studies evidenced that although both constructs are associated with empathetic dysfunction, the underlying mechanisms differ. In adults, psychopathy/psychopathic traits were associated with diminished affective empathy and intact cognitive empathy, whilst the opposite was seen autistic adults and those with elevated autistic traits. In children, those with autistic traits or a diagnosis of autism had diminished cognitive empathy, but not affective empathy, while the relationship between callous and unemotional traits/psychopathy and empathy amongst children was less clear. The co-occurrence of autism and psychopathy was seen to lead to additional empathic and cognitive impairment, but findings were mixed making it challenging to clearly describe the clinical manifestation.

**Conclusion:**

There remains a paucity of research investigating the interaction between autism and psychopathy and included studies were characterised by multiple measurement difficulties. Attention should be directed toward developing better methods for identifying psychopathic traits in autistic individuals to advance our understanding of the relationship between autism and psychopathy to allow for the development of appropriate care pathways for this population.

**Systematic review registration:**

https://www.crd.york.ac.uk/PROSPERO/display_record.php?RecordID=413672, identifier CRD42023413672.

## 1 Introduction

### 1.1 Autism, psychopathy and criminality

Autism is a neurodevelopmental disorder characterised by social and communication deficits and restricted or repetitive patterns of behaviour (1), with prevalence currently estimated as one in 100 (2). Aggression is not a core symptom of autism but rates of aggression in autistic children and adolescents range from 25% (3) to 53% (4). This aspect of autism has been growing in interest with research increasingly focusing on the relationship between autism and psychopathy.

Psychopathy is characterised by shallow emotional response, a diminished capacity for empathy or remorse, callousness, and poor behavioural control (5, 6). Prevalence in the general population is estimated at 4.5%, with a higher prevalence among offenders (7). It has long been associated with criminal and violent behaviour and is a key predictor of recidivism (8). Psychopathy can be categorised into primary and secondary psychopathy; primary psychopathy results from largely genetic and biological influences, and secondary psychopathy is related to adverse environmental factors (such as developmental trauma/maltreatment) (9). Primary psychopathy is associated with increased emotionally stability, fearlessness, and being more self-assured than secondary psychopathy, which is often associated with greater psychopathology. As children and young people are still developing, they are not considered capable of presenting with psychopathy; instead, a precursor is observed, referred to as callous and unemotional traits [CUTs; (10)].

Whilst the link between psychopathy and criminality is well evidenced (11), the relationship between autism and criminality is less clear. Collins et al. (12) reported that criminality rates amongst those with autism ranged from 0.2% (13) to 62.8% (14) within their systematic review, indicating an overrepresentation of autism amongst offenders. Despite this, the review suggested that there is little evidence that autistic individuals have an increased risk of committing crimes, highlighting methodological limitations which impacted the reliability of conclusions. It was hypothesised that social communication difficulties may make autistic individuals more likely to be viewed as risky, encounter the criminal justice system, and receive custodial sentences.

### 1.2 The role of empathy

Autism and psychopathy are both characterised by empathic dysfunction which plays a role in their behavioural phenotypes, and whilst they may appear to share surface similarities, the underlying difficulties may differ (15). Empathy involves understanding and sharing others’ emotions, thoughts or feelings and can be divided into cognitive (understanding thoughts and feelings) and affective (sharing emotional experiences) empathy (15). It has been proposed that autistic people struggle with cognitive empathy but not affective empathy, whereas the opposite is found within psychopathy (16–18).

Cognitive empathy requires theory of mind (ToM)/perspective taking skills, and together with affective empathy both are required when making moral decisions (19). Autistic people who have difficulties with cognitive empathy may inadvertently cause harm to others due to difficulty interpreting the behaviour of others (20), while individuals with psychopathy are more likely to engage in criminality and have difficulties with affective empathy and emotion recognition, but present with intact ToM skills (15, 21). Those with psychopathy are thought to have difficulties with recognising aversive emotions in others (e.g., fear and sadness) resulting from deficits in amygdala and orbital/ventrolateral frontal cortex function (22) and these difficulties interfere with learning and subsequent avoidance. For example, fearfulness is aversive, and if attenuated, an individual may behave in self-gratifying manner without concern about the consequence as they experience no fear of negative consequences for themselves or others. There is also evidence of difficulties with recognising non-aversive emotions (23) which may be related to difficulties with attention allocation to the eyes of others (24). Diminished affective empathy, paired with the ability to mentalise, enables psychopaths to successfully manipulate others for personal gain (15). This contrasts with autistic individuals who experience aversive emotions if they believe they have caused harm (20). Therefore, although both autism and psychopathy are characterised by empathic dysfunction, behaviour and decision-making are very different and driven by distinct empathetic pathways.

### 1.3 Aims and rationale

Little is known about the co-existence of autism and psychopathy. Rogers et al. (25), proposed the ‘double hit’ hypothesis, whereby autistic individuals may also show additional impairments in empathy, best explained by the presence of psychopathy as a distinct and additional disorder. However, research in this area is limited. Therefore, the aim of the current study was to systematically review the literature to: (a) understand the relationship between psychopathy and autism, and (b) to describe the clinical manifestation of the two constructs when they co-occur. Studies examining this relationship are critical in furthering our knowledge of this small but clinically significant population group and may help to inform the types of interventions appropriate for those who meet the criteria for both constructs, and especially those who encounter criminal justice as a consequence of their behaviour. The review will encompass traits of each disorder to reflect the spectrum nature of both constructs. Research on children with CUTs (considered a pre-cursor to adult psychopathy) will be included because early identification can help prevent serious risk through successful early intervention.

## 2 Method

This systematic review was conducted according to PRISMA guidelines (26) and was registered with PROSPERO (registration number CRD42023413672).

### 2.1 Search strategy

Relevant studies were identified by systematic searching of the following databases: PsychINFO; CINAHL Ultimate; Medline Ultimate. Google Scholar was also searched and backward searching of identified papers was completed. Grey literature was searched through www.opengrey.eu. Initial searches were undertaken in March 2023 and completed in April 2023. Key terms were searched using English and American terminology, spelling, and truncation to ensure that all variant word endings were identified. Search terms were combined using the term *‘AND’*, **Table 1**.

**Table 1:** Summary of search terms.

Search Category	Summary of terms
Autism	Autism Spectrum Disorder OR Autis* OR ASD OR ASC OR Asperger* OR ‘Pervasive Developmental Disorder/condition’ OR ‘neurodevelopmental disorder’ OR Kanner*
Psychopathy	psychopathy, OR psychopathic OR psychopath* OR CU traits OR *callous unemotional*

To ensure searches produced relevant results only, the above search terms were restricted to title only and a further specified term of *‘NOT psychopathology’* was included within the title or abstract. This was because initial searches without this clarification produced multiple inapplicable results. Searches were restricted to English language and academic journals or dissertations, in line with the eligibility criteria below, **Table 2**.

**Table 2:** Eligibility criteria.

Inclusion criteria	Exclusion criteria
1. Studies investigating the relationship (similarities, differences, shared variance or overlap) between symptoms, traits or characteristics of psychopathy/CUTs and ASD 2. Clinical or non-clinical sample (for example, traits of ASD and psychopathy/CU traits and not just formal diagnoses) 3. Articles written in English 4. Articles published in peer reviewed journals and/or grey matter	1. Review articles, editorial/opinion pieces, book chapters 2. Articles focusing on Antisocial Personality Disorder/Conduct Disorder or Oppositional Defiance Disorder that do not consider CUTs or psychopathy or a relationship to ASD

Due to limited research in this area, no limiters or restrictions were placed upon study design or study date.

### 2.2 Screening and article selection

**Figure 1:**
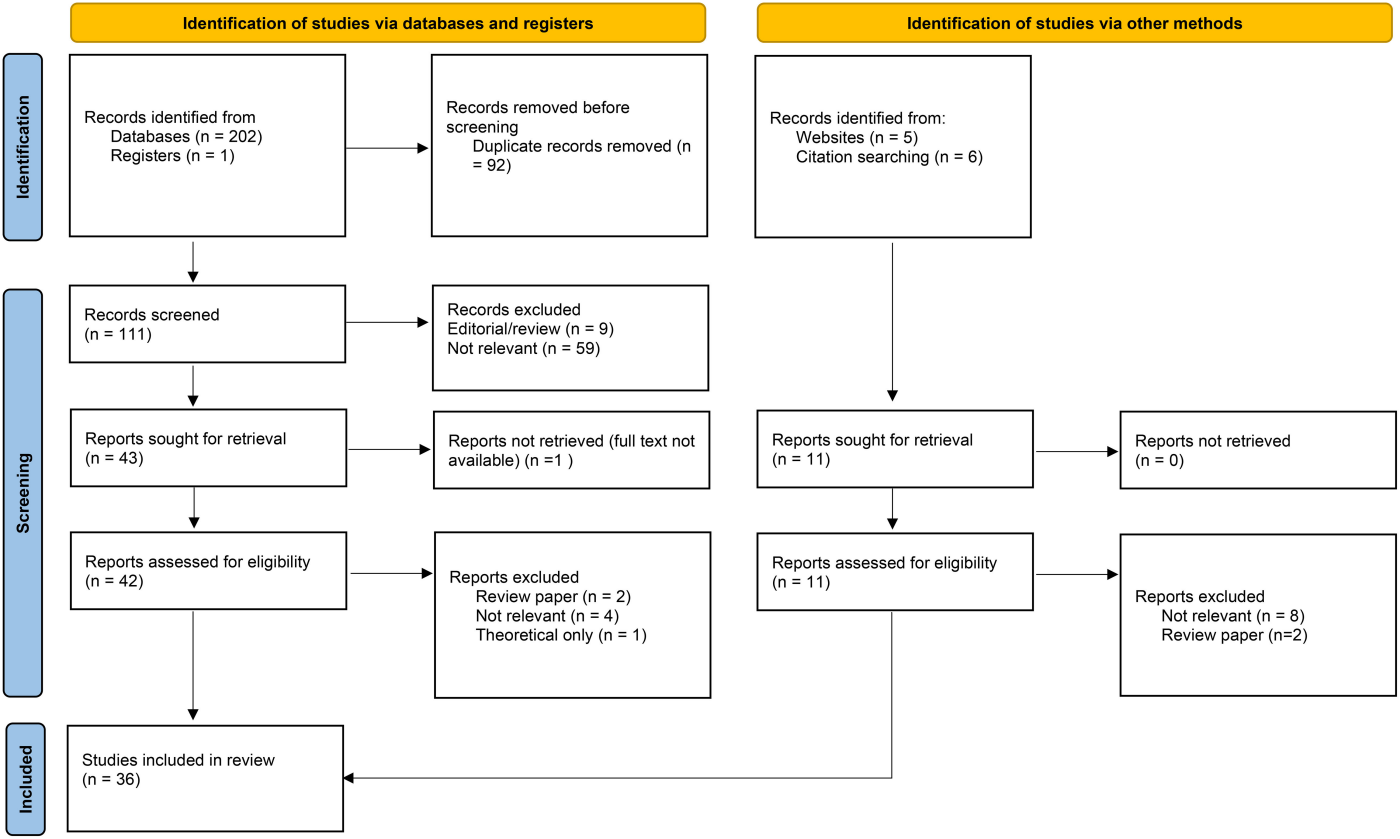
PRISMA diagram showing screening and identification of eligible studies (27).

Article selection was completed by author KM, with 30% of search results also screened by an independent, masked, second rater (HW), with an interrater agreement of 100%. Preferred Reporting Items for Systematic Reviews and Meta-Analyses (PRISMA) (26) guidance was used to refine studies and can be seen in **Figure 1** which details article selection. First, duplicates were electronically removed using EBSCO. Abstracts were then screened against the eligibility criteria and results were rejected which did not meet criteria. This included book chapters or papers not specifically looking at both autism and psychopathy in some manner. Full text screening of remaining articles was then completed.

## 3 Analysis

### 3.1 Data extraction

The following data were extracted from each paper: author and country, study population and participant characteristics, measure of autism/psychopathy/CUTs administered and main findings. These data were considered relevant to either quality appraisal of the studies or relevant for synthesis of findings in relation research question. Thirty percent of papers were checked by HW, with an inter-rater agreement of 88%. All disagreements were resolved through discussion.

### 3.2 Quality appraisal

Prior to evidence synthesis, a critical appraisal of the literature is required to enable a judgement about bias and subsequent effectiveness. Study quality was assessed using the ‘Checklist for Analytical Cross-Sectional Studies’ (28). This tool is used to assess the methodological quality of each included study and assess sources of bias. One included study (29) was a longitudinal cohort study and therefore the ‘Checklist for Cohort Studies’ was used instead (30). These tools are recognised as a reliable tool for use in systematic reviews to evaluate variation in study designs and methodology (31). Again, 30% of papers were checked by HW, with an inter-rater agreement of 82% and disagreements resolved through discussion.

### 3.3 Synthesis

A narrative synthesis approach was adopted due to the broad spectrum of included research. This was conducted in line with guidance by Popay et al. (32), who describe this technique as a synthesis of studies relying on the use of words to summarise and explain findings.

## 4 Results

### 4.1 Study settings and sample size

Of the 214 papers identified during initial searches, 92 duplicates were removed, 71 were not relevant and 13 were reviews or editorial pieces. The full text article was unavailable for one paper, and another was theoretical only, leaving 36 studies that met the eligibility criteria and were included, **Figure 1**. **Table 3** shows 22 studies that recruited children and **Table 4** shows 14 studies that included adult participants.

**Table 3:** Studies investigating the relationship between psychopathy/ callous unemotional traits and autism/autistic traits in children.

Author, country	Aim of study	Study population	Participant characteristics	Measures of Autism, Psychopathy and/or CUTs	Main Findings	Quality Rating
**Bedford et al. (**33). UK	1. Examine emotion recognition in children with high CUTs (for static and dynamic facial expressions) and if this is moderated by gaze direction. 2. Assess the impact of co-occurring autistic traits on relationship between CUTs and emotion recognition.	Sample taken from existing cohort study - Wirral Child Health and Development Study.	N = 292 (152M, 140F) Mean age – 7.25	SCQ – current (parent rated) ICU (parent rated)	- ASD and CUTs significantly positively correlated (r = .396, p <.001). - High CUTs associated with reduced emotion recognition for static angry and happy facial expressions but no impairment for sad or scared faces and no link for dynamic expressions. - Association was non-significant after controlling for autistic traits, suggesting that emotion recognition difficulties in CUT group may be partly due to autistic traits and not CUT. - No association between CUTs and looking to the eyes. - Reduced emotion recognition accuracy associated with higher autistic traits in static and dynamic expressions, with reduced looking to eyes for static expressions only.	7/8
**Bedford et al. (**29**).** UK	1. Examine if atypicality in infant regulatory functioning is specific to traits of ASD/ADHD/CUTs or a common shared factor. 2. Test if infant regulatory functioning moderates the association between known infant markers of ASD/ADHD and later disorder traits.	Sample taken from existing longitudinal cohort study of infants at familial risk of autism – British Autism Study of Infant Siblings. Low risk group recruited from volunteer database.	N = 104 High familial risk of ASD (N=54, 21M, 33F) Low familial risk of ASD (N =50, 21M, 29F) Age at time point 1: 7 - 14 months Age at time point: 2 – 7 years	AOSI ADOS-2, ADI-R SRS-2 (parent rated) ICU (parent rated)	- Reduced infant regulatory function associated with later traits of ASD and ADHD but not CUTs. As infant regulatory functioning is precursor to EF, this suggests that EF do not appear to be impaired in CUT.	10/11
**Bours et al. (**34**).** Netherlands	1. Examine common and unique eye tracking patterns of emotional face processing in individuals with either ASD or ODD/CD in comparison to TD individuals and explore possible modulatory role of CUTs, psychopathic traits and subtypes of aggression.	Recruited from institutes for juvenile psychiatry problems/behaviour problems, via Dutch federation of autism and leaflets in community.	N = 122 (M) Mean age = 15.4 Age range = 12-19 ASD, N = 50 ODD/CD, N = 44 TD, N = 28	ASD – relied on existing diagnosis, with some checked with ADOS and ADI SCQ (carer completed) ICU (parent and self rated) YPI (self rated)	- ODD/CD group scored higher on CUTs and aggressive behaviours than ASD and TD groups. - ASD and ODD/CD groups both fixated less on eye regions of emotional faces (except sadness) compared to TD group. - ASD and ODD/CD groups both took longer to first fixate on eyes of fearful faces compared to TD group, but nominal significance which did not survive multiple comparisons.	7/8
**Georgiou et al. (**35**).** Cyprus	1. Investigate effect of CUTs and/or autistic traits in predicting affective or cognitive empathy. 2. Consider age and genders effects in this relationship.	Recruited from schools and selected based on low or average to high level of empathy.	N = 163 (91M, 72F) Mean age = 7.3 Age range = 3 - 8	ICU (parent rated) SRS (parent rated)	- Positive correlation between CUTs and autistic traits (r= 0.60, p <.001). - Autistic and CUTs both uniquely negatively associated with cognitive empathy. - CUTs (not autistic traits) also associated with affective empathy deficits (remained after autistic traits controlled for). - Autistic traits moderated relationship between CUTs and affective empathy in girls only; CUTs among girls with high level autistic traits associated with decreased affective empathy. In boys with CUTs, affective empathy deficits only explained by CUTs. - No effect of age and empathy was found.	7/8
**Georgiou & Fanti (**36**).** Cyprus	1. Compare physiological reactivity in response to empathy eliciting or emotional stimuli in children aged 4-10 years, with autistic or CUTs. 2. Investigate interaction of CUTs and autistic traits in predicting physiological reactivity. 3. Explore age effects in relationship between CUTs and autistic traits and physiological reactivity.	Recruited from schools and selected based on low or average to high level of empathy.	N = 109 (61M, 48F) Mean age = 7.3 Age range = 4-10	ICU (parent rated) SRS (parent rated)	- CUTs and autistic traits moderately correlated (r = .51, p <.001) - Boys (only) with high levels of CUTs traits exhibited low skin conductance reactivity during sad and fearful stimuli. - No significant associations for females with high CUTs or autistic trait group. - CUTs associated with stronger heart rate reactivity to fear stimuli only when autistic traits were low.	5/8
**Ibrahim et al. (**37**).** USA	1. Examine shared and distinct neural signatures of emotional face perception in children with ASD, with and without disruptive behaviours and review amygdala and reactivity and connectivity with prefrontal regions.	ASD groups recruited from clinical setting. TD group recruited from community setting.	N = 57, (46M, 11F) Age range = 8-16 ASD + disruptive behaviour group, N=18 ASD group, N= 20 TD group, N=19	ICU (parent rated) ADI-R ADOS-2	-Children with ASD and disruptive behaviour have reduced amygdala and ventrolateral prefrontal cortex connectivity compared to ASD only group during emotional processing task. -CUTs did not significantly predict amygdala- ventrolateral prefrontal cortex connectivity after controlling for externalising behaviour. - CUTs and externalising behaviour associated with reduced amygdala reactivity to fearful faces in children with ASD after controlling for suppressor effects. - Neural mechanisms of disruptive behaviours in ASD could be distinct from core symptoms of ASD.	7/8
**Jones et al. (**16**).** UK.	1. Compare cognitive and affective empathy profiles in male children with psychopathic traits versus ASD.	Schools – mainstream, emotional and behavioural difficulties schools and ASD settings.	N = 96 (M) Age range = 9 – 16 Psychopathic traits, N = 21 ASD, N = 21 CP, N = 23 TD = 31	ICU (teacher rated) ASD – relied on existing diagnosis	-ASD group showed deficits in cognitive perspective taking but not affective empathy. - Psychopathic trait group showed deficits in affective but not cognitive empathy (evidenced by them caring less about victims’ feelings than other groups). - Psychopathic trait group attributed less fear to themselves than other groups.	7/8
**Jones et al. (**38**).** UK	1. Assess extent to which aetiology of psychopathic traits is independent of autistic traits. 2. Study aetiology of emotion attribution ability and its association with psychopathic tendencies and autistic traits.	Secondary data from cohort twin study – Twins Early Development Study.	N = 642 twin pairs Mean age = 9 Age range 8-10 98 pairs MZ twin boys, 89 pairs MZ twin girls, 126 pairs DZ boys, 104 pairs DZ girls, 225 opposite sex twins	APSD (parent rated) CAST short version (parent rated)	- Heritability of both psychopathy and autistic traits individually with moderate positive phenotypic association with each other. - Most genetic influences accounting for individual differences in psychopathic tendencies were unique to that domain. - Genetic and non-shared environmental influences related to psychopathy traits were unique to each phenotype, although the disorders shared some environmental influences. - Poor emotion attribution associated with higher levels of psychopathy and ASD and these associations were largely explained by common genetic factors.	5/8
**Klapwijk et al. (**39**).** Netherlands	1.Compare neural correlates of processes involved in empathy in youth with ASD, youth with CD and CUT and TD youth.	Recruited from child psychiatric clinics, juvenile detention centres/forensic psychiatric unit and through local advertising.	N = 79(M) Age range = 15-19 ASD, N =23 (mean age 17 years) CD/CU+, N = 23 (mean age = 16.6) TD, N = 33 (mean age = 17.1).	ADOS ADI-R ICU (self report)	-During emotion recognition task, boys with ASD showed reduced responses in brain areas associated with mentalising compared to other groups, suggesting a deficit in cognitive empathy. -During emotional resonance tasks, both the CD and CUT group and the ASD group showed reduced amygdala responses compared to TD group, suggesting deficits in affective resonance; however, the reduced responses occurred in different brain areas suggesting disorder specific features. - CD and CUT group showed deficits in brain area (left inferior frontal gyrus and interior insula) associated with affective empathy which the ASD group did not show.	8/8
**Leno et al. (**40**).** UK	1.Investigate Prevalence of CUTs in youth with ASD. 2. Investigate whether CUTs are associated with impairment in recognition and reduced looking to eyes for fearful faces in youth with ASD.	Sample recruited from ongoing longitudinal research - QUEST follow up study.	N = 211 (169M, 42F) Mean age – 13.51 Mean IQ – 72.5	Clinical diagnosis of ASD (partially confirmed via ADOS and ADI-R). SCQ (rater unclear) ICU (mix of shortened and full versions) (parent rated)	-22% adolescents with ASD scored above cut off for CUTs (cut off expected to identify top 6% of CUTs scores in general population). - Higher CUTs associated with lower IQ and more severe ASD symptoms. - CUTs are elevated in ASD and result in more higher conduct problems and less prosocial behaviour. - All participants demonstrated impairment in recognition of fearful faces (compared to other emotions) but no effect of ASD severity or CUTs. - CUTs in autistic sample associated with longer reaction times to identify fear and less eye contact during viewing of fearful faces.	6/8
**Leno et al. (**41**).** UK	1.Investigate prevalence of CUTs in adolescents with ASD and test association with behavioural and cognitive measures (EF, emotion recognition and ToM). 2. Investigate association between CUTs and CD in sample of adolescents with ASD 3. Test if fear recognition is associated with CUTs in sample of adolescents with ASD	Sample recruited from ongoing cohort study - Special Needs and Autism Project.	N = 92 (84M, 8F) Mean age = 15.5 Age range = 14.7-16.8 IQ > 50 Mean IQ = 84.7 Autism, N = 48 PDD, N = 44	ADI-R ADOS SRS (parent rated) APSD (parent rated)	-51% scored above cut off for CUTs (cut off expected to identify top 6% of CUT scores in general population). 17% of these had concurrent conduct problems, *vs* 9% with low CUTs (not significantly different). -ASD and elevated CUTs traits did not show elevated level of conduct problems compared to general population with CUT. - CUTs in ASD associated with specific impairment in fear recognition but not ToM or cognitive flexibility (EF skills).	6/8
**Leno et al. (**42**).** UK	1.Investigate emotion recognition ability and impact of eye gaze in sample of children enriched for social, emotional and behavioural difficulties with either autistic traits or CUTs.	Recruited from schools (including schools for children with social, emotional and behavioural difficulties), social media and charities.	N = 171 (75M, 96F) Mean age = 13.14 Age range = 10-16 ASD, N = 99	Parent reported ASD diagnosis SRS (parent rated) ICU (parent rated)	-Associations between autistic and CUTs and emotion recognition were dependent on gaze cueing. -Higher CUTs associated with lower emotion recognition accuracy (not specific to fear) in the uncued condition. Association was non-significant when controlling for conduct problems. - Fear recognition improved with cued eye gaze in high CUTs group. No improvement in other emotions. - Autistic traits associated with decreased emotion recognition in cued condition only. Association was non-significant when controlling for conduct problems.	7/8
**Parys (**43**).** USA	1. Investigate prevalence of symptoms of ASD and CUTs in adolescents in treatment for sexual offences. 2. Does measure of CUTs differ for participants with and without autism? 3. Investigate differences in emotion facial recognition for participants with and without autism.	Residential treatment programme for sex offenders.	N = 7(M) Mean age = 16 Age range =14- 19	APSD (self rated) ICU (self rated) CARS-2-HF (staff rated) SRS (second edition) (staff rated)	- 3 participants met criteria for mild to moderate ASD, within these none met criteria for CUTs on APSD but 2 did on ICU. - 4 participants did not meet ASD criteria, and within these one had CUTs according to APSD and one according to ICU, one on both measures. - No significant difference in emotion recognition, or social skills for participants with/without autistic traits. All participants lacked appropriate sexual knowledge. - Only scores for unemotional factor on ICU were different for ASD *vs* non-ASD group, with higher score for ASD group.	2/8
**Pasalich et al. (**44**).** Australia	1. Investigate additive and interactive effects of CUTs and autistic traits in relation to cognitive and affective empathy in children with conduct problems.	Recruited from child behaviour research clinic (excluding ASD diagnoses).	N =134 (106M, 28F) Mean age = 5.6 Age range = 3-9	Items combined from SDQ and APSD to assess CUTs (parent and teacher rated SRS (parent rated)	- High autistic traits independently associated with impaired cognitive empathy only. - High CUTs independently associated with impaired affective and cognitive empathy. - Marginal significant interaction found between CUTs and ASD traits and affective empathy – children with high levels of CUTs and ASD traits had most pronounced deficits in affective empathy.	6/8
**Pijper et al. (**45**).** Netherlands	1. Examine interactive and additive effects of CUTs and autistic symptoms in relation to empathy within boys with ODD/CD.	Recruited via clinical health centres and special educational schools.	N = 49(M) Mean age = 10.28 Age range = 7-12 IQ >70 ODD, N = 32 CD, N = 17	SRS (parent rated) APSD (parent and teacher rated)	- Negative association between CUTs and empathic sadness (as a measure of affective empathy). - Symptoms of autism moderated this relationship with higher levels of autistic traits showing less impaired affective empathy. - Negative association between autistic traits and cognitive empathy.	6/8
**Rogers et al. (**25**).** UK	1. Determine if psychopathic behaviour is expression of ASD or independent. 2. Assess difference between individuals with ASD and high CUTs *vs* those with ASD and low CUTs. 3. Compare cognitive data from autistic sample with high or low CUTs to previously collected data of young people with psychopathic tendencies.	Recruited from specialised residential school (for autistic students with violent or difficult externalising behaviour).	N = 28 (M) Mean age =14	SCQ (parent rated) APSD (teacher rated)	- Psychopathic traits not related to severity of ASD or related to cognitive deficits associated with ASD, e.g., mind reading/executive functioning skills (low correlation between CUTs and ASD). - Group differences for tasks underlying psychopathy but not ASD. -High CUTs group poorer at moral convention distinction and sadness recognition (no group difference for recognition of fear). -ASD and psychopathy different constructs, can occur independently of each other and can be measured independently.	5/8
**Schwenck et al. (**17**).** Germany	1. Compare cognitive and emotional empathy traits in different age groups of children with ASD, CD with elevated or low CUTs (CD+CUT or CD-CUT) and a matched TD comparison group. 2. Investigate age effects of empathy development.	Clinical sample recruited from local psychiatric services, controls recruited from general population via ads.	N = 192 (M) Mean age = 12 years 3 months Age range = 6-17 ASD group, N = 55. CD+CUT. N = 36 CD-CUT, N = 34 TD, N = 67.	ICU (parent rated) ADI-R ADOS SCQ	- Cognitive empathy difficulties in ASD group found, along with a delay in recognition of sad faces. Increased emotional affection compared to CD-CUT group. - CD+CUT group had deficit in affective empathy compared to all other groups but not emotion recognition or cognitive empathy. - All groups performed better with age on all skills (cognitive empathy, emotional empathy, and emotion recognition), however ASD group showed a decrease in recognition of sad faces.	8/8
**Svensson et al. (**46**).** Sweden	1. Explore association between psychopathic traits and CD in children with and without coexisting ADHD or ASD, in a community sample of twins.	Recruited from ongoing longitudinal twin study – Child and Adolescent Twin Study in Sweden.	N = 8762 (4453M, 4309F) Age 9 (47.8%) or 12 years (52.2%)	CPTI-SV (parent rated) A-TAC (parent rated)	- Weak corelations between measure of ASD and subscales of psychopathy measure (including CUTs) but moderately strong association between ASD and total scores on psychopathy measure (r = .38 for boys, r = .33 for girls).	5/8
**Tye et al. (**47**).** UK	1. Examine association between CUTs and EF skills and moderating effect of CUTs in ASD/ADHD (population with impaired executive functioning).	Recruited from outpatient neurodevelopmental clinics and local schools.	N = 92 (M), Age range = 8-13 Mean age = 10.8 IQ > 70 ASD, N = 19 ADHD, N = 18 ASD + ADHD, N = 29 TD, N = 26	ADI-R ADOS SCQ (parent rated) ICU (rater unclear)	- TD group had significantly lower CUT than all other groups. ASD and ADHD combined group had significantly higher CUT than ASD only group. - Enhanced conflict monitoring skills in ASD associated with presence of CUT – similar to non-ASD population with psychopathic traits. Heterogeneity in ASD within conflict monitoring group partially accounted for by presence of CUTs. - Suggests there is a form of ASD that co-occurs with high CUTs.	7/8
**Vilas et al. (**48**).** UK	1. Compare performance on self-report measure of empathy and on behavioural task of empathy accuracy in adolescents with ASD, adolescents with BD and a TD comparison group.	Recruited from secondary schools and specialised secondary schools (for ASD or behaviour).	N = 71 (37M, 34F) Mean age = 15.26 Age range = 12-17 ASD, N = 27 (23M, 4F) TD, N = 27 (7M, 2-F) BD, N = 17 (7M, 10F)	ASD – relied on existing diagnosis YPT (self reported) ICU (self-report) APSD (self-report)	-Significantly higher levels of CUTs in BD group than ASD and TD groups. - No group differences in affective empathy suggesting this is intact in ASD group. - ASD and BD groups had deficits in cognitive empathy (compared to TD group) with ASD group performing worse across perspective taking abilities (subcomponent of cognitive empathy scale). - BD group performed worse with online simulation (subcomponent of cognitive empathy scale); however this was none significant and may be due to characteristics of the BD group such as age or impulsively instead.	6/8

Note measures: ADI, Autism Diagnostic Interview (49); ADI-R, Autism Diagnostic Interview–Revised (50); ADOS, Autism Diagnostic Observation Scale (51); ADOS-2, Autism Diagnostic Observational Schedule, second edition (52); APSD, Antisocial Process Screening Device (53); AOSI, Autism Observational Scale for Infants (54); A-TAC, Autism-Tics, AD/HD and other Comorbidities Inventory (55); CAST, Childhood Autism Spectrum Test (56); CARS-2-HF, Childhood Autism Rating Scale, 2^nd^ edition, High Functioning Version (57);CPTI-SV, Child Problematic Traits Inventory- Short Version (58); ICU, Inventory of Callous-Unemotional Traits (59);SCQ, Social Communication Questionnaire (60); SDQ, Strengths and Difficulties Questionnaire (61); SRS, Social Responsiveness Scale (62); SRS-2 – Social Responsiveness Scale, 2^nd^ edition (63); YPT, Youth Psychopathic Trait Inventory (64).

Note disorders/diagnoses: ADHD, Attention Deficit Hyperactivity Disorder; ASD, Autistic Spectrum Disorder; BD – Behavioural difficulties; CD, Conduct Disorder; ODD, Oppositional Defiance Disorder; TD, typically developing.

Note other: EF, executive functioning; ToM, theory of mind.

**Table 4:** Studies Investigating the Relationship Between Psychopathy/Psychopathic Traits and Autism/Autistic Traits in Adults.

Author, country	Aim of study	Study population	Participant characteristics	Measures of ASD, Psychopathy and/or CUTs	Main Findings	Quality Rating
**Álvarez-Couto et al. (**65**).** Spain	1. Analyse the role of CUTs in relation to frequency of behavioural problems in adults with ASD and ID. 2. Study the role of CUTs in relation to behaviour problems less related to social environment (e.g. stereotyped behaviour suck as rocking).	Adults with autism and moderate to profound ID living in community (either with family or community placements).	N = 83 (59M, 24F) Mean age = 38.92 Age range = 18-58 All with ID	DiBAS-R ICU (completed by proxy)	- Significant but weak positive relationship between ASD and CUTs (r = 0.257, p = 0.025). - Low level of CUTs in this sample – none met cut off to be considered as exhibiting high CUTs. - CUTs indirectly mediated relationship between ASD severity and frequency of self-injurious behaviour and stereotypies (behaviour exhibited and directed towards individual) but not aggressive behaviour exhibited towards others.	5/8
**Barnard-Brak & Richman (**66**).** USA	1. Examine association between psychopathic and autistic traits. 2. Examine overlap and differences in psychopathy subscales among individuals with ASD. 3. Examine differences in scales related to ASD and psychopathy among those with ASD, ASD and psychopathic traits and those with neither ASD or psychopathic traits.	Community sample.	N = 723 (364M, 356F, 3 Non binary) Mean age - 48.53 IQ > 70 79% white	AQ10 (self rated) Dirty Dozen Scale (self rated)	- Small to moderate correlation between autistic and psychopathic traits (r = 0.19, p <.001). - 10% of sample met ASD cut off score (N =74), 12% of sample met cut off to indicate high psychopathic traits (n = 88). - 3% (n=22) met cut off scores for both autistic and psychopathic traits (30% of ASD group). - Individuals with high autistic traits, with or without high psychopathic traits, showed higher levels of impaired social skills. - Individuals with high psychopathy scores had significantly higher sensory sensitivity and restricted/repetitive behaviours than those with high autistic traits. - Individuals with traits of both had similar levels of restrictive/repetitive behaviours and sensory sensitivities to those with autistic traits and no psychopathic traits.	6/8
**Gillespie et al. (**67**).** UK	1. Examine interaction of psychopathic tendencies with autistic traits and the expression of positive psychotic experiences in a non-clinical adult sample whilst performing task to assess cognitive and affective ToM.	University students	N = 55, (16M, 39F) Mean age = 20 Age range =18-37	LSRP (self rated) AQ (self rated)	- Interaction of primary psychopathy traits and high autism traits resulted in decrement in cognitive ToM performance only. - Opposite was seen with interaction in psychosis and primary psychopathy. - Affective ToM negatively associated only with primary psychopathic tendencies.	5/8
**Helt et al. (**68**).** USA	1. Explore susceptibility to contagious yawning and its relationship with eye contact in individuals with high and low levels of autistic traits or psychopathy traits. 2. Explore relationship of each group to self-reported empathy.	University students	N = 97 (47M, 50F) Mean age = 21.48 Age range = 18.75 -24.58	AQ (self rated) PPI-R (self rated)	- High psychopathic individuals less susceptible to contagious yawning and itching, unrelated to eye gaze and negatively correlated with overall levels of empathy. - Individuals high in autistic traits only less susceptible to yawning and this relationship is moderated by eye gaze (participants with greater autistic traits spent less time looking at eyes). - Autistic and psychopathic trait groups have distinct empathy profiles with opposite relationships to personal distress.	6/8
**Jameel et al. (**69**).** UK	1. Compare individuals with high *vs* low autistic traits and those with high *vs* low psychopathic traits on counterfactual thinking task involving thinking about others’ mistakes and corresponding judgements of regret, guilt, and blame.	University students	Initial screening sample, N = 828 (41.4%M, 58.6%F) Mean age = 20 Subsample selected for final analyses, N = 79. High ASD group, N = 20 (10M, 10F) Low ASD group, N = 19 (9M, 10F) High Psychopathic group, N = 21 (11M, 10F), Low Psychopathic group, N = 19 (9M, 10F)	PPI-SF (self rated) AQ (self rated)	- High autistic trait group blamed characters in story more for their mistakes than low ASD group, suggesting poorer perspective taking ability. - High psychopathy group gave lower ratings for moral judgments of regret and guilt in characters than low psychopathy group. Perhaps due to them not expecting to feel regret or guilt themselves and therefore not recognising that others may feel this emotion.	6/8
**Leno et al. (**70**).** USA	1. Uncover common and unique patterns of neural responses to different types of social feedback associated with autistic and psychopathic traits in a sample of TD adults.	Community sample.	N = 31 (11M, 20F) Mean age = 23.35 Age range = 18-52	SRS (adult version) (self rated) SRP-SF (self rated)	- Psychopathy and autistic trait groups both showed atypical feedback processing. - Autistic trait group showed decreased sensitivity to social feedback with preserved feedback to non-social stimuli. - Antisocial domain of psychopathy was associated with overall decreased sensitivity to all types of feedback, however the interpersonal domain of psychopathy showed preserved processing of positive feedback with atypical responses to negatively valanced feedback. No association was found in neural responses to overall level of psychopathic traits. - No significant corelations were found between the SRS and any SRP-4-SF subscales.	8/8
**Lockwood et al. (**18**).** UK	1. Investigate whether psychopathic and autistic traits were differentially related to performance on affective resonance and cognitive perspective taking and whether alexithymia contributes to task performance.	Recruited from university participant databases and community.	N = 110 (55M, 55F) Mean age = 21.9 Age range = 18 -33	AQ (self rated) SRP-4-SF (self rated)	- Unique associations between psychopathic traits and reduced affective resonance but not cognitive perspective taking - Unique associations between autistic traits and reduced cognitive perspective taking but not affective resonance. - Alexithymic traits contributed to performance on affective resonance independently to psychopathic traits.	7/8
**Loureiro et al. (**71**).** Portugal	1. Investigate if amount of autistic traits in offenders differs from that in the general population (controlling for psychopathy and other confounding variables). 2. Investigate if autistic and psychopathic traits present independently to each other in prison and comparison group.	Forensic and community setting.	N = 211(M) Prison group, N = 101 Mean age = 37.4 Age range = 22-63 Comparison group, N = 111 Mean age = 39.3 Age range =18-63	AQ (self rated) TriPM (self rated)	- Autistic traits higher in prisoners than general population. - No correlations found between autistic and psychopathic traits (in either group) therefore suggestive that the disorders are distinct and that autistic traits are independent risk factor of imprisonment.	8/8
**Noppari et al. (**72**).** Finland	1.Investigate structural brain differences in ASD and psychopathy by comparing regional grey matter volume.	Recruited from prisons and neuropsychiatric clinic, controls – unknown.	N = 58 (M), Age range 20-47 ASD group, N = 20 (mean age = 28) Psychopathy group, N = 19, (mean age = 31) TD, N = 19 (mean age = 29).	PCL-R ADOS-2 AQ (self rated) LSRP (self rated)	- ASD and psychopathy group both have lower GVM in motor areas than control group. - Psychopathy group showed lower grey volume matter in frontotemporal areas (associated with social cognition and emotional aspect of empathy) than ASD group.	8/8
**Oliver et al. (**73**).** UK	1.Determine whether psychopathic and autistic traits are differentially associated with cognitive empathy, empathic concern, and affective sharing performance. 2. Investigate the relationship of trait anxiety on empathy in these groups.	Community sample.	N = 90 (36M, 54F) Mean age = 21.7	PPI-R (focus on cold-heartedness subscale) (self rated) AQ (self rated)	- Psychopathic traits negatively correlated with emotional empathy (empathic concern and affective sharing) and unrelated to cognitive empathy accuracy. - Autistic traits were unrelated to all measures of empathy, including cognitive empathy performance but this may have been impacted by ceiling effects in their test of cognitive empathy. - Autistic traits positively related to trait anxiety levels. No relationship with psychopathy cold heartedness.	7/8
**Skjegstad et al. (**74**).** Switzerland	1.Investigate social cognition in subtypes of psychopathy and determine level of neural overlap in social cognition impairments across psychopathic and autistic traits.	Community sample.	N = 113, (47M, 66F) Mean age 25.59 Age range = 18-40	LSRP (self rated) AQ (self rated)	- Positive correlation between autistic traits and secondary psychopathy only (*r* = .356, *p* < 0.001). - Secondary psychopathy and autistic traits shared greater commonalities (high neuroticism and trait anxiety, low extraversion) than primary psychopathy and autistic traits (low level of openness only). - Sensory processing deficits common for psychopathic and autistic traits, but in different areas. - Autistic traits associated with deficits in dorsal auditory processing streams used for communication context encoding. - Psychopathic traits associated with hypoactivity in socio-affective processing networks. - Most social processing networks impacted in primary psychopathy, contributing towards decreased affective empathy. - Intact empathic and affective neural processing but deficits in neural mirroring and mentalising in secondary psychopathy.	5/8
**Soderstrom et al. (**75**).** Sweden	1.Investigate extent to which features and problems assessed by the PCL-R correlate with DSM-IV diagnostic definitions of mental and personality disorders to identify unique features for psychopathy.	Recruited from forensic psychiatry clinic.	N = 100 (92M, 8F) Age range = 17 – 76	PCL-R ASDI	- 18% participants met criteria for a form of ASD. - PCL-R scores low, range from 0-27 points. - PCL-R total scores and factor 2 (unemotionality) and factor 3 (behavioural dyscontrol) significantly correlated with autistic traits. - PCL-R factor 1 (interpersonal) scores not correlated with autistic traits, suggesting that this factor represents discriminating feature of psychopathy as separate disorder (interpersonal deceitfulness, manipulation, grandiosity).	5/8
**Sun et al. (**76**).** Finland	1. Compare neural responses to emotional communicative signals in TD individuals versus psychopathic offenders and autistic individuals.	Recruited from prisons and neuropsychiatric clinic, controls – unknown.	N = 58 (M), Age range 20-47. ASD group, N = 20 (mean age 27.85), Psychopathy group, N = 19, (mean age 31.16), TD group, N = 19 (mean age 28.53).	PCL-R ADOS-2 AQ (self rated) LSRP (self rated)	- Somatomotor responses to vocal and facial emotional expressions were weakened in psychopathy and ASD groups compared to TD group, however deficits were most profound in psychopathy group (especially for anger). - Psychopathy associated with reduced somatomotor responses to almost all expressions (except crying) when compared to TD group. In ASD group, lowered brain activation only observed for laughter and disgust. - Reduced somatomotor mirroring seen in Psychopathy and ASD suggests they are less likely to experience emotional contagion, which plays a role in affective empathy and inhibition of violent behaviour.	7/8
**Vyas et al. (**77**).** UK	1. Compare performance on utilitarian decision-making tasks in groups of high and low autistic or psychopathic traits across situations involving extreme physical harm or everyday social harm. 2. Examine experience of discomfort and reasoning in decision making across groups.	Opportunistic sample of university students screened, and then highest and lowest 10% scores on measures of psychopathic and autistic traits recruited.	N = 828 for screening (41.4%M, 58.6%F) Mean age = 20 For task and analysis, N = 80: High ASD group, N = 20 (10M, 10F) Mean age = 20.3 Low ASD group, N = 20 (10M, 10F) Mean age = 20.3 High psychopathy group, N = 21 (11M, 10F) Mean age = 21.15 Low psychopathy group, N = 19 (9M, 10F) Mean age = 21.15	PPI-SF (self rated) AQ (self rated)	- All groups showed greater utilitarian decision making when physical harm *vs* social harm was involved. - High traits groups both reported less distress making utilitarian decisions compared to their respective low trait groups. - High psychopathy group had lower affective empathy ratings than low psychopathy group, but no difference on cognitive empathy scales. - High *vs* low autistic trait group comparison on empathy measure gave mixed results. High autistic trait group showed lower ratings in cognitive empathy’s ‘perspective taking’ measure but not in the ‘fantasy’ measure. In affective empathy, the high autistic trait group had higher ratings in the ‘personal distress’ subscale of affective empathy than the low autistic trait group, but no differences were found for ‘empathic concern’ subscale of affective empathy. - High psychopathy group, judged misdemeanours less harshly than low psychopathy group. No group differences in ASD groups. Suggests comprised moral judgement in high psychopathy group but intact in ASD groups.	6/8

Note measures: ADOS-2, Autism Diagnostic Observational Schedule, second edition (52); AQ, Autism Quotient (78); AQ10, Autism Quotient, 10 item version (79); ASDi, Asperger Syndrome Diagnostic Interview (80); DiBAS-R, Diagnostic Behavioural Assessment for Autism Spectrum Disorder-Revised (81); Dirty Dozen Scale (82); ICU, Inventory of Callous-Unemotional Traits (59); LSRP, Levenson Self Report Psychopathy Scale (83); PCL-R, Psychopathy Checklist Revised (6); PPI-R, Psychopathic Personality Inventory -Revised (84); PPI-SF, Psychopathic Personality Inventory-Short Form (85); SRP-SF, Self Report Psychopathy Scale – Short Form (86); SRS – Social Responsiveness Scale (62); TriPM, Triarchic Model of Psychopathy (87).

Note disorders/diagnoses: ASD, autism spectrum disorder; ID, intellectual disability; TD, typically developing.

Note other: GVM, grey volume matter; ToM, Theory of mind.

Studies were conducted in 11 Western countries: UK (17), USA (5), Netherlands (3), Sweden (2), Finland (2), Cyprus (2), Spain (1), Switzerland (1), Germany (1), Portugal (1) and Australia (1). Twenty studies recruited from community settings, including schools and universities, and a further five were recruited from existing cohort/longitudinal studies. Five studies recruited from clinical settings such as child behaviour clinics and six recruited from forensic settings. One study focused specifically on sex offenders (43). Sample sizes ranged from seven (43), in an unpublished thesis, to several thousand in large scale twin studies (46, 88).

### 4.2 Participant characteristics

A total of 12115 children were recruited across the included studies, including 6654 males and 5461 females. Of these, 746 had primary diagnoses of autism, autistic traits, or were identified as being at familial risk of autism, although many also had co-morbid diagnoses or additional behavioural difficulties. Three hundred and nineteen were considered to have oppositional defiant disorder, conduct disorder/problems, CUT or psychopathic traits, whilst 11032 were either identified as typically developing or no information was provided. Eighteen participants only had a diagnosis of ADHD. A total of 1888 adults were recruited across our included studies, including 1133 males, 752 females and 3 people who identified as non-binary. Of these, 163 had diagnoses of autism or had autistic traits, 80 had psychopathic traits and the remaining were either considered typically developing or the information was not provided.

Twenty-four studies included males and females, whereas 12 only recruited males. Participant age ranged from seven months (29) to 63 years (71). One study included participants with intellectual disability (65), and three studies included those with mixed ability levels: Leno et al. (41) reported a mean IQ of 84.7, Leno et al. (40) reported a mean IQ of 72.5, and Soderstrom et al. (75) reported that 17% of participants had an IQ below 70.

### 4.3 Quality appraisal

Quality appraisal ratings are found in **Tables 1** and **2**. Scores ranged from two to eight, with five fulfilling the full criteria (17, 39, 70–72). An unpublished thesis (43), scored two out of eight. This low score was due to the small sample size (N=7) meaning that the statistical analysis was judged as inappropriate, whilst there was little information on eligibility criteria, confounding variables or appropriateness of the measures used.

### 4.4 Measurement tools

Some studies involved administering a gold standard diagnostic tool to participants including the Autism Diagnostic Observation Schedule (ADOS) (89) and the Autism Diagnostic Interview-Revised (ADI-R) (50), while two studies did not confirm existing diagnoses (16, 48), although both had large sample sizes, making this a time-consuming exercise. Commonly used measures of autistic traits were the Autism Quotient (AQ), the Social Communication Questionnaire (SCQ), and the Social Responsiveness Scale (SRS). These were considered reliable and valid measures, and appropriate screening tools. Research has shown that screening tools are not entirely predictive of diagnosis (90), making it important to differentiate between autistic traits and a formal diagnosis of autism across studies.

There was large variation in the measurement of psychopathy/CUTs. Many studies used the Inventory of Callous Unemotional Traits (ICU) (59), which is a 24-item scale designed to measure CUTs in children. Whilst this is a well-researched and validated measure (see Cardinale and Marsh (10) for a review), no study has validated its use in autistic children. Several studies used this measure (34, 37, 40, 47, 65). Other researchers (16, 25, 41) administered the Antisocial Process Screening Device (APSD) (53), measuring the wider construct of psychopathy in young people, but again, this has not been validated for use with autistic children. Rogers et al. (25), acknowledged this and confirmed that the APSD positively correlated with conduct problems as expected, suggesting convergent validity.

The authors of three studies (72, 75, 76) administered the Psychopathy Check List-Revised (PCL-R) (6), which is considered to be a gold standard tool. All other studies relied on self-report measures of psychopathy, which should be viewed critically as psychopathic individuals tend to lack insight into the nature of their psychopathology (91). Additionally, using self-report measures with those known to be manipulative and deceptive increases the risk of response bias (92). Research about the reliability and validity of self-report measures of psychopathy in autistic people is lacking. There is evidence that self-report personality measures used with autistic children are questionable (93), and three of the included studies used a psychopathy self-report measure with children (39, 43, 48). Vilas et al. (48) acknowledged the limitations of this and administered multiple measures to circumvent this problem. The use of a single measure of psychopathy is advised against (91); however, only five studies administered multiple measures (34, 43, 48, 72, 76).

## 5 Autism and callous and unemotional traits in children

### 5.1 Estimated prevalence

Leno et al. (40) reported that 22% of autistic children scored above their designated cut off to indicate the presence of CUTs. However, some participants completed the full ICU measure and others a shortened version. Ideally, prevalence studies should include a representative sample and exclude any possible biases; the full ICU should have been administered to all participants, and their autism diagnosis confirmed. Two groups of researchers administered the ASPD, reporting different rates of CUTs. Leno et al. (41) reported that 51% of autistic adolescents fell into their category of high CUTs. In contrast, Rogers et al. (25) reported that their sample had a mean CUT score of 4.77, which is considered an ‘average’ CUT score. However, methodological differences between these studies make comparison challenging.

### 5.2 Autistic traits and callous and unemotional traits

Three studies (33, 35, 36) with large, mixed gender samples reported a positive correlation between CUTs and autistic traits (*r* = .40, *r* = 0.60 and *r* = .51 respectively) amongst typically developing children. Studies reporting higher correlations recruited participants based upon having either low or high empathy levels which may have inflated the correlation.

### 5.3 Autism and psychopathy

Three studies made use of samples of those with an existing autism diagnosis (25, 40, 46). Svensson et al. (46) undertook a large twin study (N = 8762), and administered the Child Problematic Trait Inventory – Short Version to index psychopathy. They reported a significant relationship between psychopathy and autism amongst boys, r = .38, and girls, r = .33, bearing in mind that there may be validity issues with their choice of measure (94). Leno et al. (40) reported that higher CUTs were associated with more severe autistic traits, lower levels of prosocial behaviour and increased conduct problems. In contrast, Rogers et al. (25) reported no relationship between CUTs or psychopathy and autism and cognitive abilities in a much smaller study of autistic boys.

### 5.4 Empathy

As expected, there was evidence that autism/autistic traits and CUTs/psychopathy in children is associated with distinct empathetic profiles. Children with autistic traits demonstrated deficits in cognitive empathy with intact affective empathy (35, 44, 45), and the same relationship was observed in children with diagnoses of autism (16, 17, 39, 48). These results appeared consistent despite the variation in the measurement of empathy and methods across studies. The relationship between CUTs/psychopathy and empathy appeared less clear; some studies reported diminished affective empathy and intact cognitive empathy (16, 17), whilst others reported diminished affective and cognitive empathy (35, 44).

Studies looking at the relationship between CUTs and autistic traits had contradictory results. While Pijper et al. (45) reported a negative association between CUTs and affective empathy in their sample of 10-year-old boys with conduct disorder as expected, the relationship was moderated by autistic traits; those with higher autistic traits and CUTs exhibited less impaired affective empathy. In contrast, Pasalich et al. (44) found that 5-year-old boys and girls with conduct disorder and high levels of both CUTs and autistic traits displayed the most pronounced deficits in affective empathy. These contradictory findings may be explained by: (a) sex differences: there is limited evidence that high CUTs and high autistic traits are associated with decreased affective empathy in girls only (35) and Pijper et al. (45) only included a sample of boys, and (b) difficulties with the measurement of empathy: both Georgiou et al. (35) and Pasalich et al. (44) used the Griffith Empathy Measure (95) and there is evidence that the affective empathy scale lacks construct validity (96). Age may also have impacted on these findings as there is evidence of improved performance with age on both types of empathy in all participants (17), as would be expected, and Pijper et al. (45) included older children relative to Pasalich et al. (44). Nonetheless, it’s worth noting that another study reported no relationship between age and empathy (35).

### 5.5 Cognitive profile

There was some evidence that psychopathy and autism are distinct constructs and the interaction of these may create a distinct cognitive profile. Bedford et al. (29) reported that reduced infant regulatory function (a precursor to executive functioning) is associated with later autistic traits but not CUTs in their longitudinal study, suggesting the two constructs are associated with differing executive functioning abilities. However, they did not include data for children older than seven years, and thus lacked information about continued development. When exploring the interaction of CUTs and autistic traits, Tye et al. (47) reported that autistic children with high CUTs exhibited enhanced conflict monitoring skills. Whilst this indicates a potentially advantageous role of CUTs on executive functioning in this group of children, the study was a small-scale preliminary study using a specific task to assess conflict monitoring, which may not be generalisable to other executive functioning skills. Two studies found that CUTs/psychopathic traits in autistic children were unrelated to the executive functioning skills associated with autism (25, 41).

### 5.6 Emotion recognition

Nine studies explored emotion recognition. Ibrahim et al. (37) reported that autistic children with CUTs displayed reduced amygdala activity to fearful faces compared to those with autism only. Conversely, Rogers et al. (25) found that all autistic children demonstrated fear recognition, regardless of the presence or absence of psychopathic traits, although this study focused on the wider construct of psychopathy (not CUTs). Results for sadness differed, with Rogers et al. (25) reporting that autistic boys with high psychopathic traits had poorer sadness recognition than those with low psychopathic traits. These studies used morphed faces (25) or still pictures (37) which may not accurately reflect how emotions are viewed during in-person social interactions. Bedford et al. (33) theorised that dynamic expressions are a more accurate representation of social interactions and compared static pictures with short video clips of people performing facial expressions. They reported that CUTs in typically developing children were associated with reduced emotion recognition for static facial expressions depicting anger and happiness. This association was not observed for dynamic facial expressions and disappeared when controlling for autistic traits. In contrast, autistic traits were associated with poorer overall emotion recognition for both static and dynamic expressions. Leno et al. (40) adapted the emotion recognition stimuli from Bedford et al. (33) and investigated emotion recognition in autistic adolescents, reporting that all participants demonstrated impairment in recognition of fearful faces with no relationship with autism severity or CUTs.

Several studies investigated the role of eye gaze on emotion recognition (33, 34, 40, 42). Bours et al. (34) reported that autistic adolescents and adolescents with CUTs both showed reduced fixations of the eye regions compared to typically developing adolescents. When considering the interaction of autism and CUTs, Leno et al. (40) found that CUTs in autistic adolescents was associated with longer times to identify fear and reduced eye contact during viewing of fearful faces. Leno et al. (42) then investigated the effect of cueing attention to the eyes in children with either CUTs or autistic traits, finding that this improved fear recognition in children with CUTs (no improvement in other emotions) but had the opposite effect on overall emotion recognition in their autistic trait group, suggesting different underlying mechanisms. However, the relationship between autistic traits, emotion recognition and gaze cueing was non-significant after controlling for conduct problems, emphasising the importance of considering co-occurring psychiatric traits.

Finally, Georgiou and Fanti (36) investigated the relationship between emotional recognition and physiological reactivity and reported that boys with CUTs exhibited low skin conductance reactivity during sad and fearful stimuli, whilst no associations were found amongst girls with CUTs or children of either gender with autistic traits. CUTs were associated with stronger heart reactivity to fear stimuli amongst children with low levels of autistic traits. They theorised that low skin conductance reflected fearlessness in children with CUTs, whilst stronger heart rate reflected thrill seeking. Unfortunately, the authors did not measure anxiety which may impact physiological responses.

## 6 Autism and psychopathy in adults

### 6.1 Prevalence

Barnard-Brak and Richman (66) looked at the prevalence of autistic and psychopathic traits amongst a community sample (N = 723) without a diagnosis of autism, finding that 10% met screening cut off to indicate autistic traits and 12% met screening cut off to indicate psychopathic traits; 30% of the autistic trait group also meet criteria for psychopathic traits. The study relied on brief self-report measures of autistic [AQ-10; (79)] and psychopathic traits =[Dirty Dozen Scale; (82)], which are not diagnostic, and findings should be viewed in the context this limitation.

### 6.2 The relationship between autistic and psychopathic traits

Several studies commented on the correlation between psychopathy and autism, with wide variation in the source of participants, measures, and methodology and all administering self-reports of psychopathic and autistic traits. In community samples, Barnard-Brak and Richman (66), reported a weak but significant positive corelation, r = .19, whilst other studies reported no significant correlation (70, 71). No correlation was found between autistic and psychopathic traits in a forensic setting (71).

On the other hand, Soderstrom et al. (75) recruited violent offenders and administered the gold standard, PCL-R, and reported a significant but small positive correlation between PCL-R total, factor two (unemotionality), factor three (behavioural dyscontrol), and autistic traits. No correlation between autistic traits and factor one (interpersonal) was found. Only one study differentiated primary and secondary psychopathy, reporting a positive correlation between autistic traits and secondary psychopathy traits only (74). All the aforementioned studies measured autistic traits, and only one study recruited adults with a diagnosis of autism and intellectual disability, observing a small but significant positive relationship between autism and CUTs (65).

### 6.3 Empathy

Many studies recruited typically developing individuals without a diagnosis of autism and grouped them according to whether they had high or low autistic or psychopathic traits, drawing comparisons. As expected, findings indicated that psychopathic traits were associated with diminished affective empathy and intact cognitive empathy (18, 73, 77) whilst autistic traits are associated with reduced cognitive empathy but not affective empathy (18, 69). Of note, these studies all recruited participants with a mean age of 20-21 years, an age at which the human brain is still developing, and therefore results may not be applicable to older adults. In one study, Oliver et al. (73) failed to find a relationship between autistic traits and all measures of empathy, but the cognitive empathy test used was subject to ceiling effects, reducing the sensitivity of this task.

Studies of emotional contagion (thought to reflect affective empathy) highlighted impairment in typically developing adults with psychopathic traits and individuals with autistic traits, with greatest impairment observed in those with psychopathic traits (68). Helt et al. (68) observed that individuals with high traits of either autism or psychopathy both showed reduced yawn contagion, but the psychopathic trait group also showed reduced contagion of itching. The relationship between autistic traits and yawn contagion was moderated by eye gaze suggesting that some of the reduced contagion was due to less time spent looking at the eyes. These findings contribute to the evidence that psychopathy is associated with diminished affective empathy to a greater extent than autism. Similar results were found in autistic adults with a diagnosis; Noppari et al. (72) recruited violent offenders with high psychopathic traits, autistic adults and a typically developing comparison group. They observed weakened somatomotor responses in both their violent offender group and their autistic group (compared to their comparison group), however the most pronounced deficits were observed in the violent offender group.

Only one study investigated the interaction of psychopathic and autistic traits in relation to empathy. Gillespie et al. (67) measured primary and secondary psychopathy traits and autistic traits amongst university students and observed diminished cognitive ToM performance in students with both high primary psychopathy traits and autistic traits, concluding that people with co-occurring traits of both constructs have additional empathy impairments. No interaction effect was seen for affective ToM, which was uniquely associated with primary psychopathic tendencies. Unfortunately, this was a small-scale study, relying on self-report measures.

### 6.4 Cognitive profile

As with children, there was evidence that psychopathy and autism have different cognitive profiles and the authors of two studies compared high and low autistic or psychopathic trait groups on cognitive processes. The first group reported that adults with high autistic traits tend to blame vignette characters for their mistakes more so than those with low autistic traits, while those with high psychopathic traits attributed lower regret and guilt to vignette characters (69). The second group investigated moral judgment, reporting that the high psychopathic trait group judged misdemeanours less harshly than the low psychopathy group, with no differences in those with high or low autistic traits, leading them to conclude that moral judgement was only affected by psychopathy (77). Although offering insight into the cognitive profiles of autism and psychopathy, neither study investigated the interaction of the two constructs, and both relied on self-report measures from university students, limiting generalisability.

Two additional studies employed brain imagining techniques in individuals with autistic or psychopathic traits. Leno et al. (70) investigated neural feedback processing of social and non-social information, reporting atypical neural feedback processing in both trait groups. Autistic traits were associated with decreased sensitivity to social feedback, whilst those with traits of the antisocial domain of psychopathy showed decreased sensitivity to all feedback and those with traits of the interpersonal domain of psychopathy showed attenuated processing of negative feedback only. Skjegstad et al. (74) reported deficits in both trait groups for socio-affective processing, but again these showed different areas of association; autistic traits were associated with deficits in dorsal auditory processing streams (used for communication context encoding), whilst psychopathic traits were associated with hypoactivity in socio-affective processing networks. This study was exploratory and lacked an *a priori* power calculation, but both studies suggested distinct neural mechanisms across these constructs. Again, these studies did not investigate the interaction of these traits, failing to shed light on the ‘double hit’ hypothesis.

Regarding the interaction of psychopathy and autistic traits, (65) investigated the mediating role of CUTs in different types of challenging behaviours in a sample of autistic adults with intellectual disability. They reported that CUTs mediated the relationship between challenging behaviours directed towards the self, but not aggressive behaviours directed towards others, therefore proposing that CUTs may have a protective role for self-directed challenging behaviours. However, results must be viewed tentatively as this was a small-scale study that looked only at frequency and not severity of behaviour amongst those with both intellectual disability and autism.

## 7 Discussion

This review sought to investigate the relationship between psychopathy and autism and what happens when they co-occur. Thirty-six studies were identified as meeting eligibility criteria, largely published within the last 10 years. The variation in methodologies, study focus, measures and samples recruited, made comparisons difficult, allowing only provisional conclusions to be drawn. Further, few studies investigated the co-occurrence of autism and psychopathy and directly investigated the ‘double hit’ hypothesis making it difficult to draw clear conclusions.

Across all ages, an increased prevalence of CUTs/psychopathy in autistic individuals or in those with high autistic traits appeared to exist relative to the general population and regardless of methodology used. Prevalence rates ranged from 22%-56%, whilst prevalence of psychopathy in the general population is estimated at 4.5% (7). It remains unclear whether autistic children are at risk of developing CUTs and later psychopathy, or whether autism and CUTs/psychopathy are similar constructs and overlap. Multiple limitations were associated with the measures used, drawing urgency to the need to develop measurement tools sensitive enough to untangle this relationship.

Generally, authors reported a positive correlation between autistic and psychopathic traits amongst children (33, 35, 36). However, the authors of one study reported no significant correlation between autistic symptoms and CUTs in diagnosed autistic boys (25). In adults, the positive relationship between autistic and psychopathic traits was generally attenuated relative to children (66, 75) or not found (70, 71). This was also observed in adults with autism and intellectual disability (65). The relationship between psychopathy and autism amongst adults and children may differ due to issues with the sensitivity of measurement tools and development; autistic and psychopathic traits will likely change with maturation.

Several papers evidenced that although the constructs are both associated with empathy dysfunction, the underlying mechanisms differ. In adults, psychopathy/psychopathic traits were generally found to be associated with diminished affective empathy and intact cognitive empathy, whilst the inverse relationship was seen in autism/autistic traits which is consistent with both theory and other research (21, 97). A recent meta-analysis confirmed that psychopathy is associated with diminished affective empathy (98). Research about autism and affective empathy is inconsistent but points towards fewer deficits in this area compared to cognitive empathy (99), with some studies reporting intact affective empathy in autistic individuals (100).

In children, autism/autistic traits were also associated with difficulties with cognitive empathy but not affective empathy while the results for those with CUTs/psychopathy were inconsistent. Some studies reported deficits in both types of empathy and others reported difficulties with affective empathy only. This inconsistency may be due to developmental maturation throughout childhood (101) or gender, as children of both genders with psychopathic traits had difficulties with cognitive empathy but there was some evidence that males overcame these difficulties during their pubertal years (102). However, the authors of one study reported no relationship between age and empathy (35), which is unexpected, whilst another reported improved performance with increasing chronological age (17); however, they included a broader age range (six to 17 years) of boys only with intact cognitive empathy, whereas Georgiou et al. (35) included younger boys and girls (three to eight years).

In the current review, the findings from studies about emotion recognition were mixed. In adults and children, CUTs/psychopathy was associated with reduced emotion experience and emotion recognition ability, in particular, recognition of fear and sadness was diminished. These deficits largely remained in the presence of autism, for example, autistic boys with psychopathic traits showed poorer sadness recognition (25), and reduced amygdala activity to fearful faces was observed in autistic children with CUTs (37). However, results were inconsistent across studies with one study reporting a non-significant association between CUTs and emotion recognition after controlling for autism (33).

Previous research has indicated that fear recognition deficits in psychopathy are associated with poor attention to the eyes, resulting in blunted affect and impaired processing of affective cues in others (103). This association has been found across many samples, including children with CUTs (24, 103), community samples (104) and psychopathic offenders (105, 106), with similar findings in the current review identified by Bours et al. (34). Regarding the co-occurrence of CUTs and autism, it appears that deficits in eye gaze remain, with autistic children with CUTs taking longer to identify fear and showing reduced eye contact when viewing fearful faces, relative to autistic children with fewer CUTs (40).

Cueing to the eyes has been shown to improve fear recognition in children with CUTs (103). This was replicated in a single study identified in the current review, but the converse relationship was found in an autistic trait group who evidenced reduced fear recognition following cueing (42). It is possible that autistic individuals view eyes as threatening or over-arousing stimuli, thus avoiding this area and missing social processing cues which then interferes with emotion processing (107). This may explain why cueing to the eyes reduced fear recognition ability in autistic individuals but not in individuals with CUTs.

With regards to the ‘double hit’, Rogers et al. (25) reported that although psychopathy and autism can co-occur, they are not part of the same construct, finding that autistic boys with CUTs have additional impairments in moral convention distinction and sadness recognition. In the current review, two studies reported increased empathy deficits in individuals with traits of both; Pasalich et al. (44) found that boys with elevated CUTs and autistic traits showed greater impairment in affective empathy and in adults, and Gillespie et al. (67) found that the interaction of autistic and psychopathic traits was associated with reduced cognitive ToM but not affective ToM. They defined cognitive ToM as the ability to infer thoughts, intentions and beliefs of another and affective ToM as the ability to understand another’s emotions. These studies offer support to the ‘double hit’ hypothesis, suggesting increased deficits when the constructs co-exist. However, contrasting results were reported by other studies which indicated that the co-occurrence of these constructs offers enhanced skills, including less impaired affective empathy (45) and greater conflict monitoring skills (47). Unfortunately, based upon the studies included with the current systematic review, it was difficult to coherently describe the clinical manifestation of co-occurring autism and psychopathy due to some mixed findings. However, our findings offer support to the suggestion that autism and psychopathy are distinct constructs which further alter the empathic ability and cognitive ability of an individual when they co-exist.

### 7.1 Strengths and limitations

In the current review, the search strategy restricted the search terms to the title only and included the specifier *‘NOT psychopathology’.* Although this was done in efforts to screen out inapplicable results, it could have potentially led to the exclusion of some studies. The inclusion of the grey literature was a strength, but only one unpublished thesis was found. It is also important to recognise the wide focus of the review as both a strength and a limitation. Whilst this allowed for inclusion of a broad range of research, the wide focus also made it challenging to draw more specific conclusions, which may have been possible by restricting the eligibility criteria. Psychopathy and autism are highly heterogeneous, and the studies recruited a broad range of participants which is perhaps reflected in the variation of results.

In terms of limitations of the included research, only two studies (67, 74) differentiated between primary and secondary psychopathy and none considered the impact of adverse childhood experiences. In psychopathy research, children with CUTs showed strongest deficits in emotion recognition when there was no history of maltreatment, suggesting that this may be a feature of the primary variant only (108). As adverse childhood experiences are common in autistic children (109), this is an important variable to consider when seeking to determine the relationship between psychopathy and autism.

### 7.2 Clinical implications

The increased prevalence of CUTs/psychopathy in autistic individuals underscores the importance of assessing psychopathy as part of the evaluation of autistic offenders or those at risk of offending to better understand their presentation. Understanding this at an early stage could lead to more targeted treatment options. The studies included within this review were characterised by multiple difficulties with measurement, including lack of validated measures for identifying psychopathic traits within autistic individuals, highlighting this as an area requiring attention. There was a lack of intervention studies, however there was some evidence to suggest that interventions to improve eye contact may be a helpful strategy to improve emotion recognition in psychopathic individuals but may have a detrimental impact for autistic individuals (42). The impact of such interventions for individuals with both psychopathy and autism is unclear but clinicians should be aware of the different underlying mechanisms and consider this with implementation of any emotion recognition strategies used.

### 7.3 Future directions

Although research in this area appear to have grown substantially since Rogers et al. (25) introduced the concept of the ‘double hit’ hypothesis, clear gaps remain. Firstly, there remains a lack of research focusing on the interaction of both autism and psychopathy which is critical in furthering our understanding of the clinical manifestation of the two constructs when they co-occur. Age and gender remain relatively unexplored variables, with fewer studies focusing on females which may be important given indicated sex differences in psychopathy (110). The presentation of primary and secondary psychopathy variants in autistic individuals is unexplored and may be important as autistic individuals experience increased adverse childhood events. Furthermore, future research would benefit from longitudinal studies exploring the developmental trajectory of autistic adults with co-morbid psychopathy or autistic children with CUT. Finally, to aid research in this area, it is essential to establish the validity of measures of psychopathy within autistic individuals, as well as the validity of measures of autism with those scoring high on measures of psychopathy. It was notable that there was a lack of studies about autistic traits amongst those with high psychopathy. These directions will all support better understanding of the relationship between psychopathy and autism and support the development of appropriate care pathways within clinical and forensic systems.

## Data availability statement

The original contributions presented in the study are included in the article/supplementary material. Further inquiries can be directed to the corresponding author.

## Author contributions

KM: Methodology, Formal analysis, Conceptualization, Writing – review & editing, Writing – original draft. HW: Formal analysis, Writing – review & editing. FB: Supervision, Writing – review & editing. PL: Writing – review & editing, Writing – original draft, Supervision, Methodology, Formal analysis, Conceptualization.
